# Transcriptome and HS-SPME-GC-MS analysis of key genes and flavor components associated with beef marbling

**DOI:** 10.3389/fvets.2025.1501177

**Published:** 2025-05-09

**Authors:** Yanling Ding, Yanfeng Zhang, Xiaonan Zhou, Chenglong Li, Zonghua Su, Junjie Xu, Chang Qu, Yun Ma, Yuangang Shi, Xiaolong Kang

**Affiliations:** Key Laboratory of Ruminant Molecular and Cellular Breeding, College of Animal Science and Technology, Ningxia University, Yinchuan, China

**Keywords:** crossbred Wagyu cattle, beef grade, flavor components, marbling, transcriptome

## Abstract

Wagyu cattle are well-known for their rich marbling. Qinchuan cattle have slower-depositing marbling than Wagyu cattle. However, because of an increase in the consumer demand for high-quality beef and the increasingly stringent standards of beef quality, improving the marbling grade of Qinchuan cattle has become particularly crucial. Therefore, we here considered castrated crossbred Wagyu cattle (crossed with Qinchuan cattle) as the research subjects. Flavor substances in the *longissimus dorsi muscle* (LDM) of A1 and A5 grades were detected through headspace-solid-phase microextraction-gas chromatography–mass spectrometry (HS-SPME-GC-MS) and electronic nose (E-nose) analysis. Fat deposition-regulating functional genes in both groups were identified through RNA sequencing (RNA-seq) and Weighted gene co-expression network analysis (WGCNA). The results showed that the intramuscular fat (IMF) was significantly higher in A5-grade beef (32.96 ± 1.88) than in A1-grade beef (10.91 ± 1.07) (*p* < 0.01). In total, 41 and 39 flavor compounds were detected in A1 and A5 grade beef, respectively. Seven aroma compounds were identified base on odor activity values (OAVs) ≥ 1, namely decanal, hexanal, nonanal, heptanol, 1-octen-3-ol, pentanol, and hexanoic acid-methyl ester. Additionally, *FABP4*, *PLIN1*, *LIPE*, *ACACA,* and *CIDEA* were the key genes primarily involved in cholesterol metabolism, sterol metabolism, and the PPAR signaling pathway in the two grades of beef. This study attempted to offer comprehensive information on marbling formation-associated candidate genes and gene-enriched pathways, which provides data for future research in beef cattle breeding and beef quality improvement.

## 1 Introduction

Beef is a nutritious and flavorful meat, and its quality and taste determine consumer requirements (1). Beef quality traits are evaluated based on shear, cooking loss, backfat thickness, and marbling. The richness of marbling is the key criterion for meat quality evaluation and is closely related to tenderness and flavor (2). The higher intramuscular fat (IMF), the marbling, tenderness, and flavor are also better in beef (3). The catabolism of key volatile compounds significantly increased in high IMF roast beef, which is consistent with a more intense sensory flavor (4). The flavor, sweetness, tenderness, and juiciness of roasted beef increased with increased marbling, whereas its sourness and astringency decreased (5). Therefore, increasing the IMF content of beef is essential for augmenting its flavor.

In flavor studies, sensory is a method for evaluating flavor. At present, the E-nose and HS-SPME-GC-MS are also frequently used to study flavor components and correct subjective judgments made during sensory evaluation. E-nose is a sensing method used for analyzing and discriminating aroma profiles and involves 10 sensors, including W1C, W5S, W3C, W6S, W5C, W1S, WIW, W2S, W2W, and W3S (6, 7). HS-SPME-GC-MS, an analytical method combining SPME and GC-MS, which is often applied in flavor studies of meat (8). In a study, ethyl-acetate, ethyl-propionate, and ethyl-hexanoate were identified in pork by HS-SPME-GC-MS. These volatile compounds were negatively correlated with pork freshness (9). Through HS-SPME-GC-MS, 31 volatile compounds were identified in yak and cattle-yak, and the contents of 2-methyl butanal, 2,3-butanedione, 1-propanediol, and 4-methyl-2-pentanone varied between the two beef type (10). In addition to HS-SPME-GC-MS, aroma thresholds and OAVs are also crucial for evaluating flavor compounds, with OAVs ≥ 1 considered to indicate key aroma compounds (11). For example, 19 key flavor compounds are involved in the grilling process of lambs (12). Furthermore, 3-methyl butyraldehyde was the key aroma compound detected in roasted chicken meat (13).

The flavor of livestock meat is chiefly influenced by sex, breed, and feeding conditions. However, differences in nutrient composition and meat flavor are observed among the same breeds under uniform feeding conditions. Genetic factors such as feed conversion efficiency and fat deposition capacity are the predominant cause of these differences. As sequencing technologies, various sequencing tools have been used to investigate genetic factors that affect meat quality. Of them, Beef eq aids in rapidly and efficiently detecting meat properties such as screening for differentially expressed genes (DEGs) in muscle, fat, and other tissues (14). RNA-seq analysis unveiled that 14 genes were differentially expressed between distinct marbling traits of *longissimus dorsi muscle* (LDM) samples from 20 Nellore cattle, and marbling formation was found to be strongly correlated with lipid and myoglobin oxidation (15). According to a functional analysis of DEGs and metabolites, *ACACA*, *PLIN1,* and *FABP4* were significantly upregulated in Pingliang Red cattle and crossbred Wagyu cattle, and the contents of 3-iodo-I-tyrosine, arachidonic acid, and cis-aconitic acid were higher in crossbred Wagyu cattle. These genes and metabolites were critical regulators and intermediate in lipid oxidation, which increased fat deposition and beef tenderness (16).

Wagyu has a high propensity to accumulate IMF (marbling), with even distribution of marbling in the meat (17). Marbling in Wagyu cattle belongs to high heritability (0.38–0.50) (18). The IMF content is lower in Qinchuan cattle than in Wagyu cattle. Crossbreeding improves animal growth, adaptability, and meat quality (19). For example, Angus × Nellore progeny were heavier at birth than Nellore progeny (20). The shear force was lower, and the myofibrillar fragmentation index was higher in Aberdeen Angus × Nelore than in Nelore (21). However, studies exploring meat flavor and molecular markers affecting marbling deposition in Wagyu × Qinchuan cattle are fewer. Therefore, in this study, the F1 generation of Wagyu × Qinchuan cattle crossbreeding was considered as the research target. The LDM of A1 and A5-grade marbling were selected to detect flavor compounds. The key aroma compounds were identified on the base of OAVs ≥1. RNA-seq was performed on the A1 and A5-grade beef samples to screen DEGs affecting marbling formation. Using RNA-, modules correlating with the key aroma compounds were constructed, and IMF deposition-regulating core genes in beef cattle were screened through protein–protein (PPI) analysis. The present study provides new insights for improving beef marbling and meat flavor to satisfy the increasingly demanding market requirements.

## 2 Materials and methods

### 2.1 Sample collection

Thirty healthy castrated crossbred Wagyu cattle (crossed with Qinchuan cattle, age: 28–30 months) with similar weights (680.53 kg ± 30.78 kg) were randomly selected from the farms in the Beijing region, which had maintained uniform feeding management conditions. The cattle were electrocuted and humanely slaughtered after being starved for 12 h. The cattle were slaughtered according to the GBT19477-2018 cattle slaughtering procedures. Professionally certified technicians rated marbling in beef portions collected between the 12th and 13th rib of the left half-carcass from 30 crossbred Wagyu cattle. The marbling was rated according to the Japan Meat Grading Association marbling score standard (22). At the end of grading, the LDM tissues of the A1-grade (*n* = 3) and A5-grade (*n* = 3) cattle were randomly collected, vacuum-sealed, and stored at −20°C for estimating IMF, dry matter content, and other indices, or were stored at −80°C for RNA-seq.

### 2.2 Determination of flavor components

#### 2.2.1 E-nose analysis of flavor components

LDM tissues (3 g) were kept in a 50 mL headspace vial and incubated for 40 min at 25°C. Then, flavor components in these tissues were identified using the PNE3.5 E-nose (PEN3.5 Airsense, Schwerin, Germany). Supplementary Table S1 lists the components of the E-nose. The assay procedure is consistent with that of Guan et al. (23).

#### 2.2.2 HS-SPME-GC-MS analysis of flavor components

Aroma compounds were analyzed using a GC-MS system (GC-MS 2010 plus, SHIMADZU; Kyoto, Japan) equipped with a DB-WAX capillary column (30 m × 0.25 mm × 0.25 μm, Agilent Technologies; Santa Clara, CA, USA). The SPME fiber of 50/30 μm DVB/CAR/PDMS (Supelco, PA, USA) should be aged before the aroma compounds are extracted. The A1 and A5-grade LDM samples (2 g) were placed in a 15 mL headspace bottle. Then, 2-dichlorobenzene (4 μL, 6.42 μg/mL) was added to each sample as an internal standard. After the mixture was vortexed, the vessel was sealed with a PTFE membrane and placed in a water bath at 60°C for 20 min. Subsequently, the SPME fibers were inserted into a sealed extraction vial for adsorption and extraction for 20 min and immediately transferred to the gas chromatograph (GC) inlet for 5 min of desorption at 250°C. The GC conditions were as follows: the initial temperature was maintained at 40°C for 3 min, ramped to 200°C at a rate of 5°C/min, and again ramped to 230°C at a rate of 10°C/min. Helium was used as the carrier gas at a 2 mL/min flow rate, and the front inlet temperature was 250°C. The MS conditions were as follows: the ion source was electron impact at 70 eV, and its temperature was 230°C, the mass spectrometry (MS) transfer line temperature was 280°C, the solvent delay was 3 min, and a full-scan mode was adopted across 50 to 500 m/z.

#### 2.2.3 Content analysis of flavor components and OAVs

The flavor components were identified by referring to the NIST14 database, retention index (RI) reference values, and authentic volatile standards (24). The content of each volatile in the varying beef grades was calculated based on the o-dichlorobenzene peak area at a known mass concentration. The OAVs were employed to evaluate the contribution of volatile aroma components to the overall beef flavor. OAVs ≥ 1 were considered to indicate the key aroma components for the A1 and A5-grade beef (25). Supplementary Table S2 lists the calculation methods for flavor compounds and key aroma components.

### 2.3 Measurements of beef quality traits

Using a straightedge, backfat thickness was measured at the 10th and 11th ribs of the carcass. The eye muscle area was the LDM cross-sectional area at the penultimate 1 and 2 thoracic vertebrae positions of the carcass. The eye muscle area was traced using sulfate paper and the cross-sectional area was calculated as follows: height (cm) × width (cm) × 0.7.

#### 2.3.1 Dry matter content analysis

The weighing flask was placed at 105°C until a constant weight was attained and weighed using an electronic weighing scale. This weight was recorded as W0. Then, the meat sample was completely defrosted and chopped into mince. Subsequently, 3 g of the minced meat sample was weighed into a weighing flask and recorded as W1, after which they were placed at 105°C until a constant weight was attained and weighed. This weight was recorded as W2. The dry matter content was calculated using Carvalho et al.’s method (26), as follows: (W2 – W0)/W1 × 100%.

#### 2.3.2 IMF content analysis

The IMF content in the LDM was extracted using the Soxhlet extraction method (27). The meat sample was dried at 105°C and weighed accurately at approximately 0.7 g. This weight was recorded as W. The sample was then wrapped in a filter paper and baked at 105°C until a constant weight was achieved, cooled, and weighed. This weight was recorded as W1. The filter paper was placed in a Soxhlet extractor and extracted with anhydrous acetaldehyde for 8 h. Then, the paper was removed, dried at 105°C for 2 h, and weighed. This weight was recorded as W2. The crude fat content was calculated as follows: (W1-W2)/W × 100%.

#### 2.3.3 Crude protein analysis

The crude protein content was analyzed using a fully automatic Kjeldahl nitrogen determination instrument (Kjeltec, FOSS). The determination standard was referred to as GB/T 9695.11-2008.

#### 2.3.4 pH_24h_ analysis

After the LDM was refrigerated at 4°C for 24 h, the electrode tip of an acidimeter was inserted into the center of meat samples of different grades and allowed to stand for 5 min, and then, the data were read. The pH_24h_ of each sample was determined three times. The pH meter was calibrated with pH 4.0 and 7.0 buffers at 4°C before use. During measurements, the pH meter was recalibrated using standard buffers that corresponded approximately to the temperature of the muscle to compensate for the effect of temperature on pH readings.

#### 2.3.5 Cooking loss analysis

The cooking loss was analyzed according to Musundire et al.’s method (28). First, 100 g of meat samples of different grades were weighed using an electronic scale, and this weight was recorded as W1. Then, all meat samples were simultaneously kept in a thermostatic water bath at 90°C for 45 min, removed, cooled to room temperature, dried, and weighed. This weight was recorded as W2. The cooking loss was calculated as follows: (W1 – W2)/W1 × 100%.

### 2.4 mRNA preparation and sequencing

RNA was isolated from A1 (*n* = 3) and A5 (*n* = 3) grade LDM by Trizol (Invitrogen, Carlsbad, CA, USA). The integrity of the isolated RNA was examined using a Bioanalyzer 2100 system (Agilent Technologies, Santa Clara, CA, USA). Magnetic beads with oligo (dT) were used to enrich mRNA after total RNA quality was assessed. Using purified mRNA as a template, single-stranded cDNA was synthesized with a random hexamer primer. Then, DNA polymerase I and RNase H were added to synthesize double-stranded cDNA. Using the Illumina HiSeq2000 platform, the cDNA libraries were bipartite sequenced by Chidio Biotechnology Co., Ltd. (Guangzhou, China).

### 2.5 Sequencing data analysis

After the data were sequenced, the raw data were filtered using FastQc v0.1. The clean data were mapped to the bovine reference genome (Bos_taurus.ARS_UCD1.2.new.genome.fa) by using Hisat2 v2.2.1 (29). StringTie v2.1.2 was used to assemble the mapped results (30). The gene expression levels of A1 and A5 groups were compared using DESeq2 v1.20, with |Fold Change| ≥ 2 and *p* < 0.05 established as thresholds of significant difference between the groups. The functional and pathway enrichments of DEGs were analyzed using clusterProfiler v4.0.0 (31). *p* < 0.05 denoted significant enrichment.

### 2.6 WGCNA analysis

Coexpression modules were built using WGCNA R v1.69 (32). Thresholds were determined using “PickSoft Threshold,” and genes were clustered using TOM values. Genes having similar expression trends were grouped into modules, each containing at least 50 genes. Similar modules were merged with a threshold of 0.25, and genes in coexpressed modules were then identified. Pearson correlation between gene expression and key flavor substances was further determined. The correlation between gene significance (GS) and module membership (MM) was analyzed, with GS > 0.9 and MM > 0.9 being considered as thresholds for identifying the hub genes in the modules.

### 2.7 PPI analysis

PPI networks were constructed using STRING v11.5 and visualized using Cytoscape v3.8.0 (33). The core genes were screened using MCODE and CytoHubba plugins in Cytoscape.

### 2.8 RT-qPCR analysis

In total of 10 DEGs were selected to verify the accuracy of RNA-seq results through RT-qPCR. Total RNAs were extracted and assayed for concentration, and then were reverse transcribed into cDNA according to PrimeScript™ RT reagent Kit (Takara, Kyoto, Japan) with gDNA Eraser to remove genomic DNA. The first step of reverse transcription including 2 μL 5 x gDNA Eraser Buffer, 1 μL gDNA Eraser, 2 μL total RNAs, and 5 μL RNase-Free ddH_2_O. Then, water bath at 42°C for 2 min. After that, 1 μL PrimeScript RT Enzyme Mix I, 1 μL RT Primer, 4 μL 5 x PrimeScript Buffer 2, and 4 μL RNase Free ddH_2_O were mixed with the reaction solution in the first step. The reaction procedure was 37°C for 15 min, and then 85°C for 5 s. RT-qPCR was performed according to a previous method of our laboratory (34). Supplementary Table S3 lists all primer sequences. All samples contained 3 biological replicates and 3 technical replicates. All data were expressed as Mean ± SEM.

## 3 Results

### 3.1 Meat quality traits analysis

Differences in the eye muscle area and cooking loss between the A1 and A5-grade beef were nonsignificant (*p* > 0.05). Backfat thickness and IMF were higher in the A5-grade beef than in the A1-grade beef (*p* < 0.01). Crude fat increased significantly in the A5-grade beef than in the A1-grade beef (*p* < 0.05). By contrast, dry matter content, crude protein, and pH_24h_ were lower in the A5-grade beef than in the A1-grade beef (*p* < 0.05) (Table 1).

**Table 1:** Meat quality features in two marbling groups.

Meat quality traits	A1	A5
Backfat thickness/mm	15.93 ± 0.32	22.77 ± 0.65**
Eye muscle area/cm^2^	41.00 ± 2.87	51.67 ± 3.85
IMF content/%	10.91 ± 1.07	32.96 ± 1.88**
Dry matter content/%	43.59 ± 1.56	34.06 ± 1.63*
Crude protein/%	59.42 ± 0.86	40.72 ± 4.96*
pH_24h_	5.72 ± 0.08	5.46 ± 0.05*
Cooking loss/%	21.35 ± 1.56	23.63 ± 0.58

*p < 0.05, **p < 0.01. All data in the table were Mean ± SE.

### 3.2 E-nose analysis of flavor components between two marbling groups

**Figure 1:**
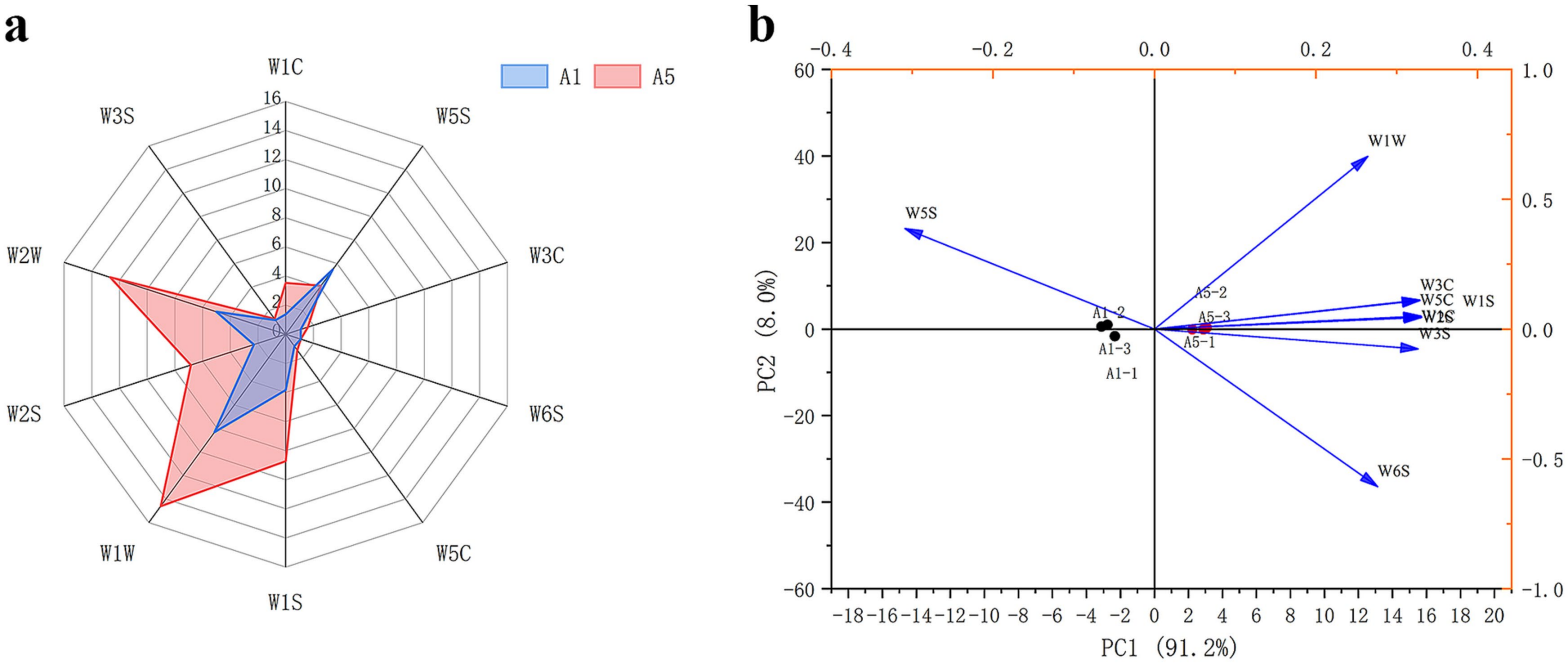
Radar diagram **(a)** and PCA **(b)** for flavor compounds in A1 and A5 grade beef.

E-nose radar fingerprinting revealed that the W3S, W1C, W3C, W6S, and W5C sensors displayed lower responses in both A1 and A5-grade beef samples, which indicated that alkanes, ammonia, aromatic compounds, and olefins exert less influence on meat quality. By contrast, the sensors W5S, W1S, W1W, W2S, and W2W displayed increased responses in both A1 and A5-grade beef samples. Furthermore, the responses of W1W, W2W, W1S, and W2S were higher in the A5-grade beef than in the A1-grade beef. This indicated that the content of alcohol compounds was higher in the A5-grade beef (Figure 1a).

The PCA analysis was performed using the E-nose data at 100 s for A1 and A5-grade beef. The results demonstrated that the PC1 and PC2 total contribution rate reached 99.2%, thereby exhibiting good ability to distinguish volatile compounds in the A1 and A5-grade beef. According to the PCA, W1W, W3C, W1C, and W1S were correlated with the A5-grade beef, and W5S was correlated with the A1-grade beef (Figure 1b).

### 3.3 HS-SPME-GC-MS analysis of flavor components

Through HS-SPME-GC-MS, 55 flavor compounds were evaluated in the A1 and A5-grade beef, including 9 aldehydes, 14 alcohols, 4 ketones, 7 acids, 2 alkenes, 9 alkanes, 6 esters, and 4 other compounds. Of them, 41 flavor compounds were identified in the A1-grade beef and 39 were identified in the A5-grade beef (Table 2). Based on OAVs ≥ 1, both A1 and A5-grade beef were found to contain 7 key aroma compounds, including three aldehydes, three alcohols, and one ester. Additionally, the contents of decanal, hexanal, heptanol, and 1-octen-3-ol were higher in the A5-grade beef than in the A1-grade beef (Table 3).

**Table 2:** Comparison of flavor compounds in A1 and A5 grade beef.

Group	Compound name	T	Formula	Flavor content (μg/kg)	
				A1	A5
Aldehydes (9)	2,5-Dihydroxybenzaldehyde	-	C7H6O3	2.40 ± 0.08	23.16 ± 0.90*
	2- Decyl aldehyde	0.01	C10H18O	-	4.64 ± 0.10
	Trans-2-undecenal	-	C11H20O	-	2.50 ± 0.12
	Trans-2-octenal	3	C8H14O	1.05 ± 0.06	-
	Decanal	1	C10H20O	5.35 ± 0.96	7.69 ± 0.06*
	Hexadecanal	-	C16H32O	-	7.26 ± 0.01
	Hexanal	4.5	C6H12O	14.96 ± 4.92	21.38 ± 0.44*
	Nonyl aldehyde	1	C9H18O	48.19 ± 3.54	41.64 ± 1.41*
	Capryl aldehyde	0.7	C8H16O	15.49 ± 1.47	15.83 ± 1.74
Alcohols (14)	Butyl alcohol	500	C4H10O	4.31 ± 0.22	13.22 ± 0.49*
	Heptanol	3	C7H16O	3.95 ± 0.47	20.74 ± 1.50*
	n-Hexanol	250	C6H14O	8.60 ± 0.16	32.61 ± 2.09*
	Octanol	110	C8H18O	12.30 ± 0.68	27.63 ± 1.87*
	3-octanol	25	C8H16O	39.34 ± 1.25	139.45 ± 2.94*
	Pentanol	0.86	C5H12O	29.84 ± 3.23	16.33 ± 2.18*
	1-Penten-3-ol	1	C5H10O	-	5.67 ± 0.14
	1-Undecanol	-	C11H24O	-	7.09 ± 0.73
	2,3-Butanediol	-	C4H10O2	9.96 ± 0.47	11.65 ± 0.79*
	2-Decen-1-ol	0.4	C10H20O	-	14.10 ± 0.10
	3,5-Dithiahexanol 5,5-dioxide	-	C4H10O3S2	-	10.69 ± 0.33
	3,6,9,12,15-Pentaoxanonadecan-1-ol	-	C14H30O6	0.94 ± 0.02	-
	2-Phenoxyethanol	-	C8H10O2	6.82 ± 0.54	-
	Heptaethylene glycol	-	C14H30O8	1.82 ± 0.53	-
Ketones (4)	2,3-Octanedione	78	C8H14O2	19.56 ± 2.19	38.67 ± 1.15*
	6-Methyl-5-hepten-2-one	15	C8H14O	-	10.31 ± 2.19*
	6,10-dimethylundeca-5,9-dien-2-one	-	C13H22O	2.16 ± 0.20	-
	Acetyl	-	C4H8O2	134.74 ± 4.68	98.16 ± 3.23*
Acids (7)	Acetic acid	-	C2H4O2	10.06 ± 0.72	9.62 ± 1.09
	Heptanoic acid	3,000	C7H14O2	1.22 ± 0.13	29.42 ± 1.48*
	Caproic acid	3,000	C6H12O2	12.14 ± 0.05	-
	Palmitic acid	9,000	C16H32O2	11.65 ± 3.77	-
	Nonanal acid	3,000	C9H18O2	2.69 ± 0.20	8.22 ± 2.45*
	Octanoic acid	3,000	C8H16O2	3.00 ± 0.36	3.39 ± 0.08
	Valeric acid	3,000	C5H10O2	1.23 ± 0.02	-
Alkenes (2)	Cyclooctatetraene	-	C8H8	63.85 ± 3.07	-
	3,7,11,15-Tetramethyl-2-hexadecyl acetate	-	C20H40	-	1.07 ± 0.09
Alkanes (9)	Cyclohexane, 1-(1,1-dimethylethyl)-4-methylene	-	C11H20	4.90 ± 0.72	-
	Decane	-	C10H22	10.65 ± 0.66	-
	Hexacosane, 9-octyl	-	C34H70	10.99 ± 1.45	-
	Hexadecane	-	C16H34	9.43 ± 0.48	-
	n-Pentadecane	-	C15H32	-	3.06 ± 0.70
	n-Tetracosane	-	C24H50	419.80 ± 5.10	5.57 ± 0.23*
	11-Decyltetracosane	-	C34H70	4.75 ± 1.27	7.59 ± 0.26
	Tetracontane	-	C44H90	181.11 ± 1.55	-
	n-Tridecane	-	C13H28	4.42 ± 0.77	-
Esters (6)	Beta-butyrolactone	88	C4H6O2	7.99 ± 0.03	12.17 ± 0.38*
	Phenyl carbamate	-	C7H7NO2	-	4.50 ± 0.36
	Methyl heptadecanoate	-	C18H36O2	0.41 ± 0.05	-
	Methyl caproate	27	C7H14O2	61.35 ± 1.21	97.57 ± 7.27*
	n-Caproic acid vinyl ester	14	C8H14O2	-	7.21 ± 0.05
	Methyl lactate	180	C4H8O3	49.70 ± 0.91	31.07 ± 0.21*
Others (4)	1,2-Dimethylbenzene	450	C8H10	17.62 ± 0.79	65.91 ± 11.93*
	N, N-Dimethylacetamide	-	C4H9NO	-	13.80 ± 1.47
	2-Pentylfuran	6	C9H14O	-	6.13 ± 1.89
	Ethylbenzene	2,205	C8H10	16.71 ± 2.12	27.97 ± 1.09*

“-” indicates flavor compounds not being detected, “T” indicates the aroma threshold, and “*” indicates a significant difference (*p* < 0.05). All data in the table were mean ± standard error.

**Table 3:** Key aroma compounds in A1 and A5 grade beef.

Aroma components	Aroma description	T	Flavor content (μg/kg)		OAV	
			A1 A5		A1 A5	
Decanal	Smell of lemon	1	5.35	7.69	5.35	7.69
Hexanal	The smell of grass and fruits	4.5	14.96	21.38	3.32	4.75
Nonanal	Smell of fruits	1	48.19	41.46	48.19	41.46
Heptanol	The smell of grass and herbal	3	4.31	20.74	1.44	6.91
1-Octen-3-ol	The smell of herbal and mushroom	25	39.34	139.45	1.57	5.58
Pentanol	The smell of bread, fruits, and wine	0.86	29.84	16.33	34.70	18.99
Hexanoic acid, methyl ester	Smell of pineapples	27	61.35	97.57	2.27	3.61

“T” indicates the aroma threshold.

### 3.4 Evaluation of RNA-seq data

**Figure 2:**
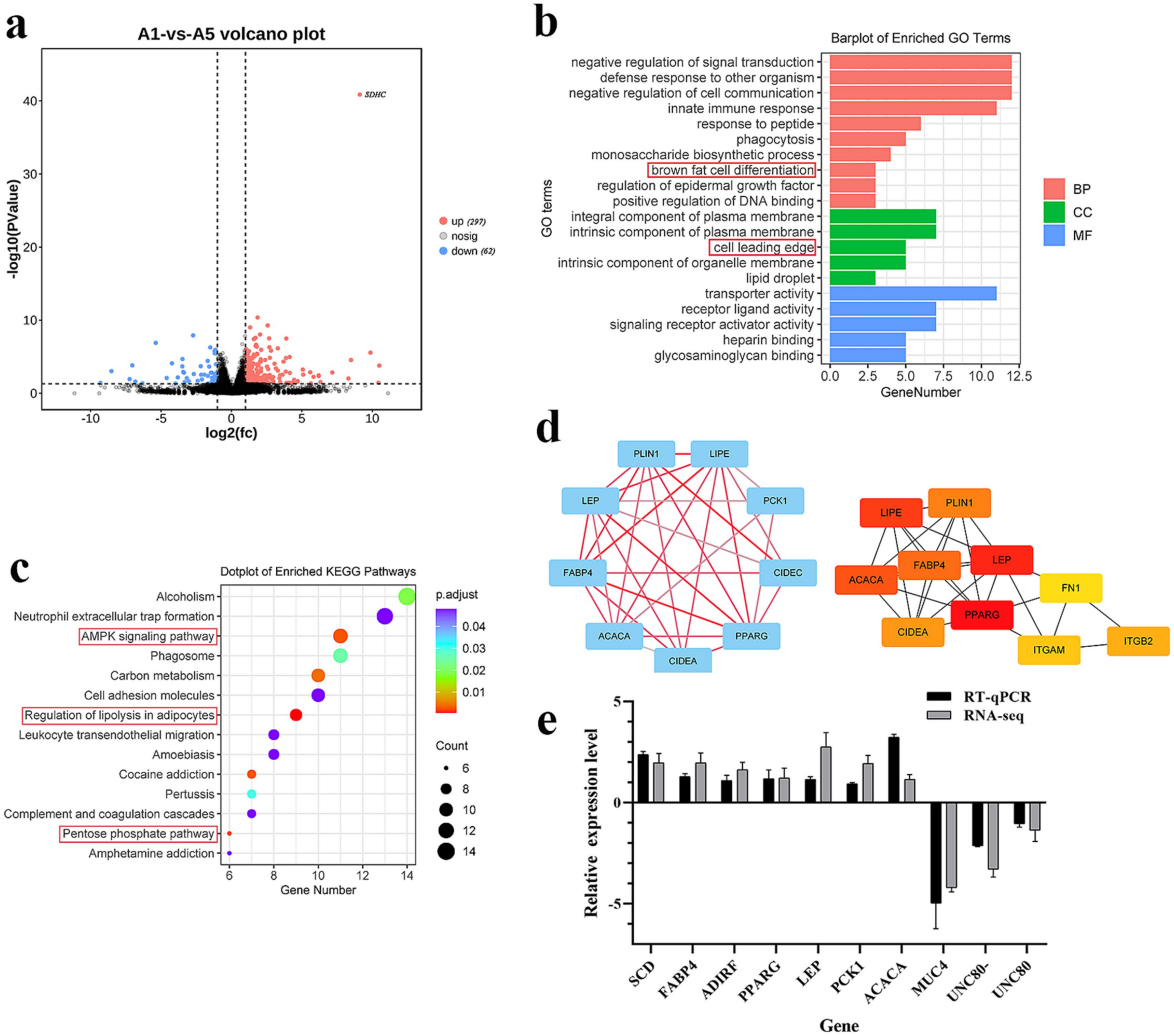
Enrichment analysis of DEGs. **(a)** Volcano map of DEGs (the red dot indicates up-regulated DEGs). **(b)** GO terms analysis of DEGs (BP indicates a biological process, CC indicates cellular component, MF indicates molecular function). **(c)** KEGG pathway analysis of DEGs. **(d)** PPI analysis of DEGs. e: RT-qPCR verification of DEGs. Data were represented as Mean ± SE. *n* = 3.

To determine the genes that affect meat quality, we performed RNA-seq analysis of the LDM of the A1 and A5-grade beef. Supplementary Table S4 lists the specific information of the RNA-seq data. Furthermore, compared with the A1 group, 297 DEGs increased and 62 DEGs decreased in the A5 group (*p* < 0.05) (Figure 2a; Supplementary Table S5). Brown fat cell differentiation and lipid droplet were the key enriched GO terms (Figure 2b; Supplementary Table S6). Moreover, DEGs were primarily enriched in AMPK and pentose phosphate signaling pathways (Figure 2c; Supplementary Table S7). The PPI results unveiled that *FABP4*, *PLIN1*, *LIPE*, *ACACA*, *LEP*, and *CIDEA* were the hub genes (Figure 2d). To verify the accuracy of the RNA-seq results, 10 DEGs were validated through RT-qPCR. Based on the results, DEGs expression levels were consistent with those in the RNA-seq results (Figure 2e).

### 3.5 WGCNA analysis

**Figure 3:**
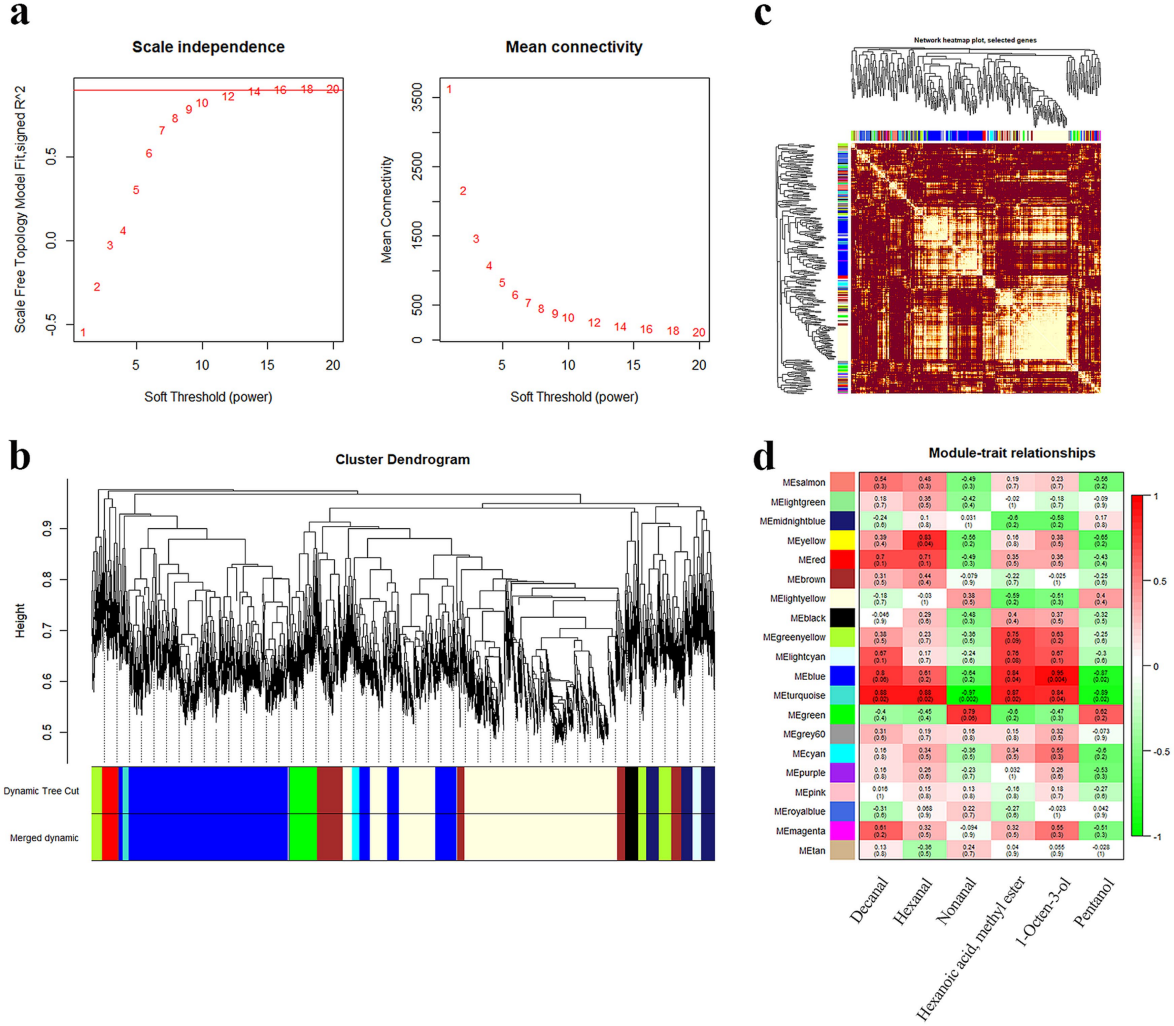
Correlation analysis of modules and traits. **(a)** Screening of optimal soft thresholds. **(b)** Co-expressed gene modules clustering tree and modules delineation. **(c)** Co-expression gene modules correlation heat map. **(d)** Heatmap of key aroma compounds and module correlation.

For WGCNA, 0.9 was the correlation coefficient threshold, and 12 was the soft threshold power (Figure 3a). Twenty co-expression modules were constructed. The largest module (light yellow) contained 1962 genes, whereas the smallest module (light cyan) contained 79 genes (Figure 3b). The heatmap displayed that these modules were mutually independent (Figure 3c). The constructed gene co-expression modules were linked to the aroma components. According to the results, the turquoise, blue, and yellow modules were related to nonanal (*r* = −0.97, *p* = 0.002), 1-octen-3-ol (*r* = 0.95, *p* = 0.004), and hexanal (*r* = 0.83, *p* = 0.04), respectively (Figure 3d).

**Figure 4:**
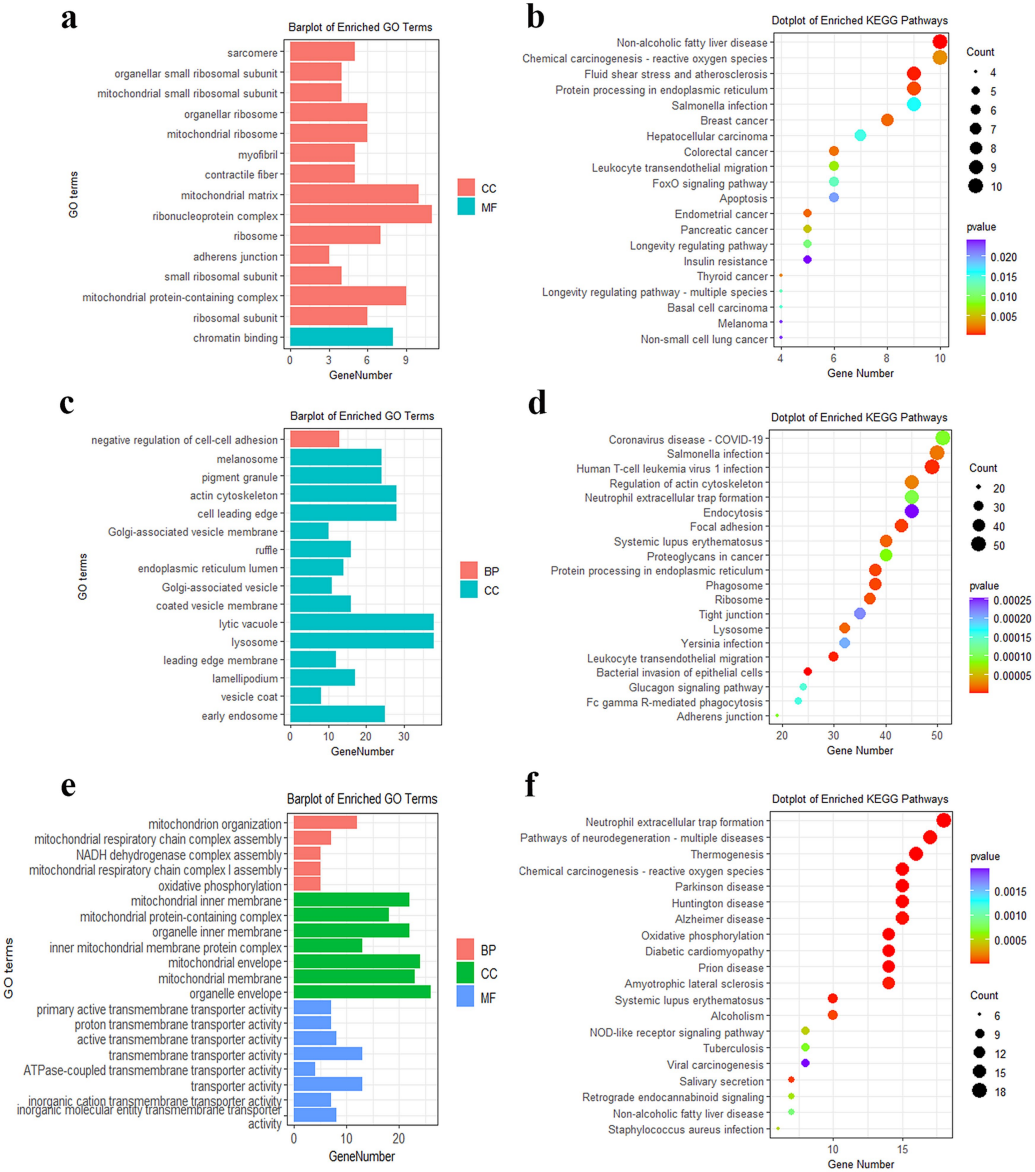
Functional enrichment analysis of DEGs in modules. **(a)** GO analysis of DEGs in the turquoise module. **(b)** KEGG analysis of DEGs in the turquoise module. **(c)** GO analysis of DEGs in the blue module. **(d)** KEGG analysis of DEGs in the blue module. **(e)** GO analysis of DEGs in the yellow module. **(f)** KEGG analysis of DEGs in the yellow module.

The turquoise, blue, and yellow modules, respectively, contained 325, 1938, and 155 genes (Supplementary Table S8). The GO term analysis demonstrated that genes in the turquoise module were significantly enriched in the mitochondrial small ribosomal subunit and mitochondrial matrix (Figure 4a). Furthermore, the 325 genes participated in insulin resistance, apoptosis, and FOXO signaling pathways (Figure 4b). The 1938 DEGs in the blue module were prominently concentrated in the negative regulation of cell–cell adhesion and actin cytoskeleton (Figure 4c) and principally involved in glucagon signaling pathways (Figure 4d). The 155 DEGs in the yellow module were chiefly involved in GO terms such as mitochondrion organization, NADH dehydrogenase complex assembly, oxidative phosphorylation, and actin cytoskeleton (Figure 4e). KEGG revealed that these DEGs were also mainly enriched in oxidative phosphorylation, NOD-like receptor, and FOXO signaling pathways (Figure 4f).

### 3.6 Functional analysis of the hub gene

**Figure 5:**
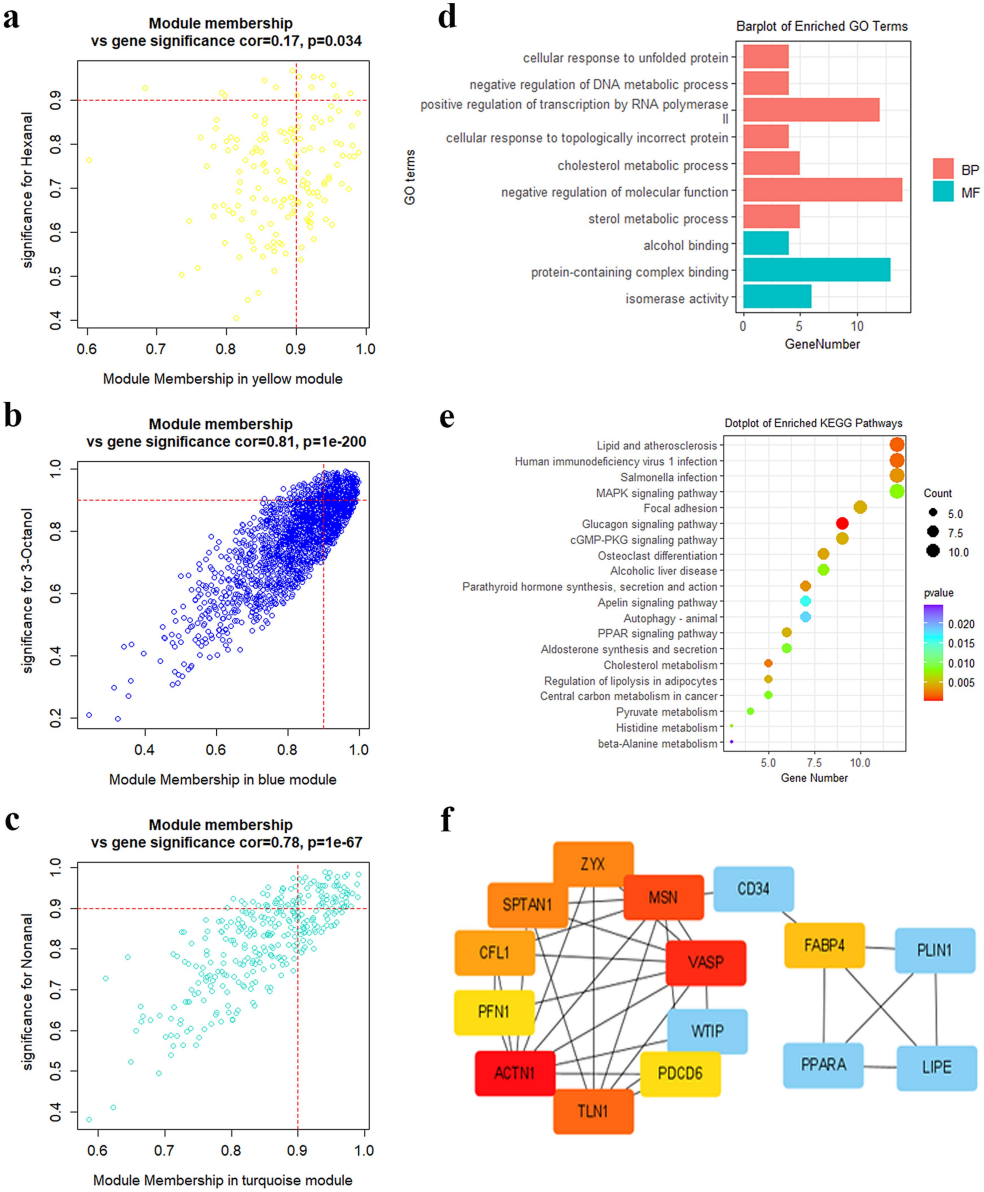
Hub gene screening and functional analysis. **(a)** Genes scatterplot in the yellow module. **(b)** Genes scatterplot in the blue module. **(c)** Genes scatterplot in the turquoise module. **(d)** GO enrichment of hub genes. **(e)** KEGG analysis of hub genes. **(f)** PPI analysis of hub genes.

In this study, hub genes in the turquoise, blue, and yellow modules were screened using GS > 0.9 and MM > 0.9. In total, 8 hub genes were identified in the yellow module. These 8 genes exhibited a correlation of 0.17 (*p* = 0.035) between GS and MM (Figure 5a). A total of 277 hub genes were identified in the blue module, and the correlation was 0.81 (*p* = 1e-200) (Figure 5b). In total, 66 hub genes were detected in the turquoise module, and the correlation was 0.78 (*p* = 1e-67) (Figure 5c). Supplementary Table S9 presents the hub genes in the three modules. According to the enrichment analysis, the hub genes primarily participated in cholesterol and sterol metabolism (Figure 5d). The KEGG results unveiled that the hub genes were predominantly concentrated in lipid synthesis and lipid metabolism signaling pathways, such as MAPK and PPAR signaling pathways (Figure 5e). The PPI analysis revealed that *FABP4*, *PLIN1*, *PPARA*, *VASP*, *MSN*, *ACTN1*, *TLN1*, and *CD34* were the core genes in DEGs (Figure 5f).

## 4 Discussion

The beef flavor is determined by its texture and aroma (35), with volatile flavor components contributing to aroma component formation (36). These components are formed through lipid oxidation, Maillard reaction, and thermal degradation (37). The key by-products of lipid oxidation are aldehydes, alcohols, and acidic compounds, including 1-octen-3-ol, nonanal, octanal, and hexanal (38). Pyrazines and furans are mainly produced through Maillard reactions, and thermal degradation and lipid oxidation contribute to flavor compound formation from non-volatile water-soluble lipids (39). Several volatile flavor compounds have been detected in livestock, such as alcohols, aldehydes, acids, hydrocarbons, and heterocyclic compounds (40). The IMF content increased significantly with an increase in beef grade (41, 42). Similarly, the content of volatile flavor compounds increased (43), including alcohols and aldehydes with a fresh flavor, ketones with an oily flavor, and esters with a milky flavor. Most alcohol compounds have specific flavors, such as 1-octen-3-ol has a mushroom flavor, octanol has a lemon flavor, and nonanol has a grassy flavor. Octanal, nonanal, and decanal have a sweet flavor, fatty flavor, and sweet flavor, respectively (44). Arginine, citrate, glucose, propionate, 3-hydroxybutyrate, and lipids are correlated to marbling in crossbred Wagyu cattle (45). Propionic acids were converted to glucose through the TCA cycle. This glucose is then available for fatty acid synthesis, which results in IMF deposition (18). The fatty content in lamb was positively correlated with flavor. Additionally, flavor compounds, such as aldehydes and alcohols, IMF content, juiciness, and tenderness were significantly higher in pork with carcasses of higher quality grades than in pork with carcasses of lower grades (46). In the present study, the IMF content was significantly higher in the A5-grade beef (32.96 ± 1.88) than in the A1-grade beef (10.91 ± 1.07). In addition, the aldehydes, ketones, alcohols, aromatic, acids, and lipid compounds were rich between A5 and A1 grade beef. Further, aldehydes, ketones, alcohols, acids, and lipids were greater in the A5-grade beef. This was concordant with the aforementioned findings, and the difference in the content of these flavor substances was the key reason for the richer marbling and IMF content of the A5-grade beef.

Meat mostly attains its flavor from volatile compounds, which rely on the IMF content (47). Fats were precursors for flavor substance formation. These substances could produce aldehydes, ketones, acids, and alcohol compounds through hydrolysis, pyrolysis, oxidation, and the Maillard reaction (48). In addition to feeding, breeding, and sex, key genes and molecular regulatory pathways are crucial factors influencing IMF deposition. RNA-seq revealed that the core genes affecting marbling and IMF content in Nellore cattle were chiefly *PLIN1*, *CISH*, *UFM1*, and *TSHZ1* (49). *FABP4*, *TPI1*, *ACTA1*, and *MDH2* were highly expressed in marbling-rich meat in Korean cattle (50).

The present study results were similar and revealed that *FABP4*, *PPARG*, *ACACA,* and *PLIN1* were highly expressed in the A5-grade marbling beef of the crossbred Wagyu cattle. *PLIN1* is a member of the lipid family that augments lipid formation (51). When adipocytes begin to catabolism, *PLIN1* modulates the activity of hydrolytic enzymes in lipid droplets to complete hydrolysis (52). Additionally, *PLIN1* was strongly expressed in subcutaneous adipose tissues, with its SNP being related to IMF content and thoracic depth in Qinchuan cattle (53). *FABP4* is an intracellular lipid chaperone abundantly expressed in adipocytes and macrophages, which can modulate lipid fluxes, transportation, esterification, and *β*-oxidation and regulate the lipid signal transduction and metabolism (54). Lipids are oxidized to produce hydroperoxides, which are then further oxidized to volatile lipid products by enzyme catalysis, including 3-hexenal, propionaldehyde, and trans-2-hexenol (55). Furthermore, 3-iodo-L-tyrosine, 2,6-diamino hexanoic acid, cis-aconitate, and arachidonic acid were positively associated with marbling, unsaturated, and tenderness (16). In lipogenesis, *FABP4* participated in the PPAR signaling pathway as an up-regulated protein, which regulates lipogenesis in human skeletal muscle cells (56). *PLIN1* was highly expressed in porcine adipose tissue and was significantly enriched in the PPAR signaling pathway (57). In summary, these genes and metabolites were pivotal factors involved in marbling formation. They are essential for enhancing fat deposition and marbling richness in beef muscles.

In this study, volatile flavor compound-related three modules were determined through WGCNA. The yellow module was positively and notably correlated with hexanal, and the blue module was significantly and positively correlated with 3-octanol and methyl hexanoate. However, the turquoise module exhibited a significantly negative connection with nonanal and pentanol. The modules were screened by determining gene-to-module correlation and gene significance. The results revealed that 8, 277, and 66 hub genes were obtained in the yellow, blue, and turquoise modules, respectively, and primarily included *PPARG*, *CD34*, and *FABP4*. Hub genes were concentrated in the MAPK, cGMP-PKG, and PPAR signaling pathways, and were involved in cholesterol metabolism. PPAR is a major pathway that regulates lipid metabolism, adipogenesis, energy homeostasis, cell growth, and differentiation (58). *PPARG* is a core regulatory gene of the PPAR signaling pathway. It was a major regulator of adipocyte differentiation (59), and a key regulator of lipid metabolism in adipocytes (60). Improving *LPL*, *FABP4,* and *PLIN1* expression activates *PPARG,* thereby driving fat deposition (61). The MAPK signaling pathway is among the major intracellular pathways for muscle development and adipogenesis (62). In a study, Qiangguyin inhibits adipogenic differentiation through the p38 MAPK signaling pathway, thereby reducing fat accumulation in OVX mice (63). Altogether, the genes *FABP4*, *PLIN1,* and *PPARG* directly or indirectly regulate IMF metabolism through molecular pathways and therefore influence the content of volatile flavor compounds. However, since the samples we collected could only satisfy the minimum number of biological replicates for RNA-seq. The results still need to be further confirmed in subsequent experiments. Meanwhile, the complex mechanism of marbling deposition at the cellular level must also be further verified.

## 5 Conclusion

In summary, the present study preliminarily elucidated flavor differences in different marbling grade beef and relevant genes affecting IMF deposition. Regarding flavor indicators, the contents of key aroma substances such as heptanol, 1-octen-3-ol, hexanoic acid, and methyl ester were higher in the A5-grade beef than in the A1-grade beef. Moreover, *FABP4*, *PLIN1*, *PPARG*, and *ACTN1* were potential candidate genes for regulating IMF deposition. These genes were enriched in cholesterol metabolism, cGMP-PKG, MAPK, and PPAR signaling pathways. The present study unveils the reasons for flavor differences in beef of different grades at the molecular level. The finding provides deeper insights into molecular regulatory mechanisms occurring during beef marbling.

## Data Availability

The data presented in the study are deposited in the NCBI repository, accession number PRJNA1009594.
