# Supplementary material for: Transcriptome and HS-SPME-GC-MS analysis of key genes and flavor components associated with beef marbling
Source: Front Vet Sci. 2025 May 9;12:1501177. doi: 10.3389/fvets.2025.1501177 (PMC12098558; doi:10.3389/fvets.2025.1501177)
Supplement: Supplementary file 1 [file Data_Sheet_1.zip › Supplementary/Table S1 The sensor array of the E-nose.docx]

Table S1. The sensor array of the E-nose

| Sensor | General Description |
| --- | --- |
| W1C | Sensitive to aromatic compounds, benzene |
| W5S | Highly sensitive to nitrogen oxides |
| W3C | Sensitive to aromatic compounds, ammonia |
| W6S | Sensitive to hydrogen |
| W5C | Sensitive to olefin, short-chain aromatic compounds |
| W1S | Sensitive to methyl |
| WIW | Sensitive to sulfur compounds |
| W2S | Sensitive to alcohols, aldehydes, and ketones |
| W2W | Sensitive to aromatic components, organic sulfides |
| W3S | Sensitive to long-chain alkanes |
