# Supplementary material for: Transcriptome and HS-SPME-GC-MS analysis of key genes and flavor components associated with beef marbling
Source: Front Vet Sci. 2025 May 9;12:1501177. doi: 10.3389/fvets.2025.1501177 (PMC12098558; doi:10.3389/fvets.2025.1501177)
Supplement: Supplementary file 1 [file Data_Sheet_1.zip › Supplementary/Table S2 The calculation methods for flavor compounds and key aroma components.docx]

The calculation methods for volatile compounds:

$$M_{x}=\frac{4*1000*A_{x}}{2*A_{i}*1000}*C_{i}$$

Ax and A_i_ represent the peak areas of the target and internal standard compounds, respectively; C_i_ represents the mass concentration of the internal standard compound (µg/mL); 1000 on the numerator represents 1 kg and 1000 on the denominator represents 1000 µL; Mx is the amount of the target compound (µg/kg).

The calculation methods for OAVs:

$$OAV=\frac{C_{i}}{T_{i}}$$

C represents the relative content of aroma components (μg/kg); T indicates the aroma threshold (μg/kg).
