# Supplementary material for: Transcriptome and HS-SPME-GC-MS analysis of key genes and flavor components associated with beef marbling
Source: Front Vet Sci. 2025 May 9;12:1501177. doi: 10.3389/fvets.2025.1501177 (PMC12098558; doi:10.3389/fvets.2025.1501177)
Supplement: Supplementary file 1 [file Data_Sheet_1.zip › Supplementary/Table S3 Primers sequences.docx]

Table S3. Primer information

| Gene |  | Primer sequence（5'-3'） | Length/bp |
| --- | --- | --- | --- |
| SCD | F | ATGGCGTTCCAGAATGACG | 107 |
|  | R | AAGAAAAAGCCACGTCGGGA |  |
| FABP4 | F | ACAGGAAAGTCAAGAGCATCG | 159 |
|  | R | TCTCATAAACTCTGGTGGCAGT |  |
| ADIRF | F | AGAAGCAGGGCAGAAAGCCAT | 100 |
|  | R | CCCAAAACCCGAGAAAGTCTCA |  |
| PPARG | F | GACCACTCCCATGCCTTTGA | 108 |
|  | R | AACCATCGGGTCAGCTCTTG |  |
| LEP | F | CATCTCACACACGCAGTCCG | 113 |
|  | R | AGATCGCCAATGTCTGGTCC |  |
| PCK1 | F | CATTGGGAACGCACACTCAC | 93 |
|  | R | ATGCCGAGGTTCCCTTTCTC |  |
| ACACA | F | CGGCTGACTGGAGTTGAAGAA | 223 |
|  | R | TCCACTTCCAAAAAGAACTCAGA |  |
| MUC4 | F | CAACCGTCCATCAAGAGCTTC | 173 |
|  | R | TGGTGCCGTTGAGGGTTTAG |  |
| UNC80 | F | TACCAGGAACAAGGCACCAAA | 271 |
|  | R | GTGCGATTTCTGCCAACCTCAT |  |
| AQP4 | F | ACTCAGCATCGCGACTATGG | 226 |
|  | R | ATTTCCGTGAACCGTGGTGA |  |
| GADPH | F | TCGGAGTGAACGGATTCGGC | 82 |
|  | R | ATGGCGACGATGTCCACTTT |  |
