# Supplementary material for: Transcriptome and HS-SPME-GC-MS analysis of key genes and flavor components associated with beef marbling
Source: Front Vet Sci. 2025 May 9;12:1501177. doi: 10.3389/fvets.2025.1501177 (PMC12098558; doi:10.3389/fvets.2025.1501177)
Supplement: Supplementary file 1 [file Data_Sheet_1.zip › Supplementary/Table S4 RNA-seq data statistics.docx]

Table S4. RNA-seq data statistics

| Sample | Raw reads | Clean reads | Q30(%) | Mapping ratio(%) | GC content |
| --- | --- | --- | --- | --- | --- |
| A1_1 | 111554164 | 111194184 | 93.56 | 94.20 | 53.84 |
| A1_2 | 101269128 | 100892594 | 92.88 | 94.08 | 54.73 |
| A1_3 | 96917862 | 96549646 | 92.69 | 93.86 | 55.06 |
| A5_1 | 90769326 | 90410038 | 93.00 | 93.80 | 56.75 |
| A5_2 | 96145566 | 95822408 | 93.13 | 93.34 | 56.20 |
| A5_3 | 91064624 | 90728852 | 93.25 | 94.34 | 54.86 |
